# Integration of experiences of contingency in patients with advanced cancer supported by a multimodal art approach

**DOI:** 10.1371/journal.pone.0319918

**Published:** 2025-04-08

**Authors:** Yvonne Weeseman, Michael Scherer-Rath, Nirav Christophe, Henny Dörr, Mirjam Sprangers, Esther Helmich, Niels van Poecke, Hanneke van Laarhoven

**Affiliations:** 1 Department of Medical Oncology, Amsterdam University Medical Centers, University of Amsterdam, Amsterdam, The Netherlands; 2 Cancer Center Amsterdam, Treatment and Quality of Life, Amsterdam, The Netherlands; 3 Faculty of Philosophy, Theology and Religious Studies, Radboud University, Nijmegen, The Netherlands; 4 HKU University of the Arts Utrecht, Utrecht, The Netherlands; 5 Department of Medical Psychology, Amsterdam UMC Location, University of Amsterdam, Amsterdam, The Netherlands; 6 Amsterdam Public Health, Mental Health, Amsterdam, The Netherlands; 7 Amsta Healthcare Organization, Amsterdam, The Netherlands; Helwan University, Faculty of Art Education, EGYPT

## Abstract

To support patients with cancer in a palliative treatment phase with the integration of experiences of contingency into their life narrative, we developed a multi-modal approach: In Search of Stories (ISOS). ISOS consists of the following elements: filling out the self-report RE-LIFE questionnaire, drawing of Rich Pictures, and reading an exemplary story with a spiritual counselor, followed by a co-creation process with a professional artist. In the current article we illustrate how patients moved through the process of integration of experiences of contingency during the meetings of ISOS by presenting two case descriptions. All meetings of the first two patients who completed the ISOS project were audio recorded, imported into Atlas-Ti and analyzed by applying a phenomenological approach to deepen our understanding of the patient’s experiences throughout the meetings. The two cases showed distinct differences on how the experience of contingency was dealt with and how the integration of experiences of contingency into the life narrative unfolded. Patients focused on life goals and values concerning connection with loved ones, and on leaving a legacy behind, which was expressed through creating a work of art. The current study provides preliminary insight into how patients can go through a process of integration of experiences of contingency into their life narrative, which could inform the development of future support for patients with advanced cancer dealing with experiences of contingency. Specifically, offering patients possibilities to express themselves through materials within an artistic setting could support these patients to find new words and additional non-linguistic ways of expressing their experiences, and thereby facilitate the integration of experiences of contingency into their life narrative.

## 1. Introduction

Living with cancer in a palliative treatment phase can be experienced as a confrontation with the randomness of life, which may profoundly disrupt one’s life narrative [1,2]. This disruption can create an interpretation crisis where existential questions such as ‘what is the meaning of my life?’, or ‘how do I relate to my own mortality?’, are brought to the forefront of one’s mind. Patients may start to doubt the fundamentals of their own life, including important life goals [2–8]. Such disruptions, here named ‘experiences of contingency’ [9,10], must be integrated into one’s life narrative to fulfill the human need for experiencing coherence, meaning and understanding of one’s self and the world [1,2]. Existential questions influence how patients appraise and react to situations and how they live their life [11]. When existentially challenged, patients indicate that they would like to address existential questions with professional support from a spiritual counselor [12]. Telling stories about one’s life may help to place life events into an intelligible whole [13] by creating causal links between those life events [14]. Talking about one’s life narrative is thought to improve patients’ quality of life [15,16].

Yet, being able to discuss existential questions is one of the unmet needs of patients with advanced cancer in the palliative phase of treatment [17,18]. To meet these needs in patients, we developed the multimodal In Search Of Stories (ISOS) approach [19]. Originating from a religious philosophical background, ISOS allows for a phenomenological approach where the impact of life events and accompanying negative emotions form the basis for the unfolding of new perspectives on one’s life narrative [19,20]. ISOS contains some elements that could also be used in psychological interventions such as life review [21,22] or reminiscence [23]. Yet, ISOS was not developed as a psychological intervention, i.e., ISOS does not treat psychopathology, nor does it specifically aim to facilitate a transition from a dysfunctional state to a more functional one. Rather, ISOS aims to support people to broaden their view and come to new action perspectives. This translates both into the integration of experiences of contingency into the life narrative and simultaneously into the development of new, emerging possibilities to live their life in a meaningful way in accordance with their ultimate life goals [19,20].

Patients are partnered up with a spiritual counselor and a professional artist. ISOS consists of the following elements: filling out the RE-LIFE – a self-report questionnaire [24], drawing of Rich Pictures [25], and reading an exemplary story with a spiritual counselor [19], followed by a co-creation process [26]. The elements of ISOS are integrated during a final meeting with the spiritual counselor and all elements are intended to initiate exploration of the life narrative and to support the process of integration of experiences of contingency.

The creation of a professional work of art within ISOS can be placed within a more recent development where art has been used to express images and impressions of cancer to support laypeople and patients in their understanding of cancer [27,28]. The works of art created within ISOS have been used in exhibitions conveying patients’ stories to a broader public [29].

Previous qualitative analysis of the co-creation part of ISOS on a select subset of patients showed that these patients discussed aspects of their life narrative during active participation in the art making process. Their life goals and the experience of contingency they reflected upon, became explicit during the process of art making and were expressed in the work of art [20]. This process of integration of experiences of contingency entailed four distinct phases. In phase 1, Art communications, patients explore various art forms through their senses. For instance, selecting significant wild flowers. During phase 2, Element compilation, initial artforms are created, for instance various compilations with wild flowers symbolizing a uterus with and without cancer. In phase 3. Consolidation, the final work of art is created, such as a protective blanket containing prints of the flower compilations. Finally, in phase 4, Reflection, patients reflect on both the work of art and the process of creating the work of art [20]. Furthermore, the process showed similarities to concepts used within resonance theory; 1. Being affected, where the patient becomes intrinsically interested in art materials, 2. Self-efficacy, inviting the patient to respond to art materials, 3. Adaptive transformation, where a work of art is created, and 4. Uncontrollability, including the entire artmaking process containing elements beyond one’s control, which might confront patients with the contingent nature of life [30]. Yet, we have not gained insight into the unfolding of the process of the integration of experiences of contingency into the life narrative throughout the entire ISOS project.

In this article, we describe how the process of the integration of experiences of contingency unfolded during the meetings of ISOS. We present two case studies that illustrate how patients moved through the meetings with the spiritual counselor, discussing the completed RE-LIFE, Rich Pictures and the chosen story, and the co-creation meetings with the professional artist. Additionally, we also include the patient’s reflection on the ISOS project as expressed during the evaluation meeting with the researcher.

## 2. Materials and methods

### 2.1. Research design and participants

**Fig 1:**
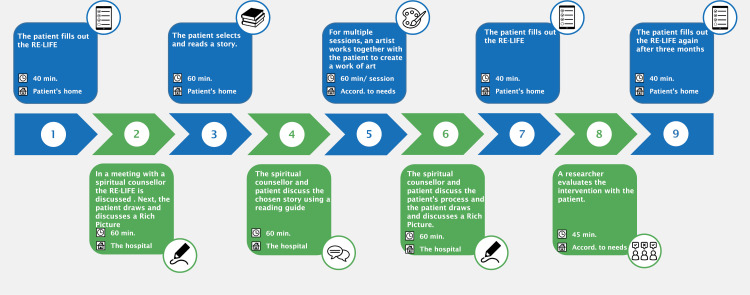
Timeline of the ISOS project.

This study has a qualitative design [19]. The elements of ISOS are explained in detail in paragraph 2.2. In Fig 1 the timeline of ISOS is presented. Numbers (1–9) of the timeline correspond to numbers used in the text. After receiving information about the project and enrollment, patients started with 1) filling out the RE-LIFE (Reconstruction of Life Events) questionnaire [24] at home. They subsequently 2) had a meeting with a spiritual counselor where they discussed their responses to the RE-LIFE questionnaire, drew a Rich Picture, and discussed the Rich Picture [25]. Then, 3) patients chose and read a story from selected literature, using a reading guide [19]. The reading guide also served as a template for 4) the second meeting with the spiritual counselor to read, discuss and reflect on the story together. Next, the patients were matched with an artist who fitted their preferences for art modalities best. In the subsequent phase of ISOS, patients and artists went through 5) a co-creation process consisting of several art making meetings, where they collaboratively created a work of art while reflecting on the patient’s experiences of contingency [20]. Once patients had finalized the co-creation process, they 6) had a third meeting with the spiritual counselor where they discussed the process to date, drew a second Rich Picture, and discussed the Rich Picture with the spiritual counselor. Following this meeting, 7) the patient completed the RE-LIFE questionnaire for the second time. Then, 8) a researcher interviewed the patients on their experience of the process as a whole. Three months after finalizing the co-creation process, 9) patients filled out the RE-LIFE questionnaire for the third time.

For the ISOS project, patients were recruited between 1^st^ March 2021 and 1^st^ August 2022, at four Dutch hospitals by their attending oncologist or nurse. Inclusion criteria were: (i) having an age of 18 years or older, (ii) receiving palliative cancer treatment, (iii) being diagnosed with metastasized cancer not more than three years prior to enrollment, (iv) being able to participate in the entire project, i.e., having sufficient cognitive ability and emotional (i.e., excluding psychopathology), verbal and physical capacity, and a life expectancy beyond the planned duration of the ISOS project. For this article we chose the first two participants who completed ISOS [31]. In total 25 patients were included in the ISOS project. Due to a high mortality rate only nine patients finalized the project, i.e., including filling out the third RE-LIFE questionnaire three months after finishing the co-creation process. Four spiritual counselors were recruited through the four participating hospitals. Inclusion criteria were: (i) having extended experience with spiritual care for palliative patients with advanced cancer, and (ii) having interest in research and collaboration with other disciplines. Eleven artists were recruited through the University of the Arts Utrecht (HKU). Inclusion criteria were: (i) having extended experience with co-creative processes with various patient groups, and (ii) being able to combine their main art modality with other art modalities, enhancing their repertoire of working with patients [26].

As ISOS addresses existential questions, special effort was taken to ensure a high level of professionalism and support for patients. The research board consisted of a multidisciplinary team covering spiritual counseling, medical psychology, medical oncology and elderly care, and was further supported by two senior faculty members of a university of arts. The daily execution of the project was overseen by the head of medical oncology and an associate professor of theology and spiritual care, who also was a certified supervisor. Within ISOS, if needed, the spiritual counselors could provide additional support for patients and could refer patients to a psychologist in the patient’s hospital. Spiritual counselors had a MA degree and extensive experience in supporting patients with existential questions and meaning making, both in a hospital setting and at patients’ homes [32]. Understanding of ethical issues, power differences, confidentiality and ability for referral to another discipline, i.e., a psychologist, is part of their professionalism. Ten of the eleven selected artists were lecturers at Universities of Arts in The Netherlands. Artists had regular supervision meetings with two senior faculty members of the University of Arts Utrecht, in which concerns regarding patients’ participation could be addressed, i.e., optimizing possibilities for patients’ self-expression and the development of the artistic process. The spiritual counselors regularly met with the main researcher to discuss progress and potential difficulties with patients. After the first two meetings with patients, the spiritual counselors together with the main researcher and both senior faculty members, established whether, from an ethical perspective, it was feasible for the patient to continue with the co-creation part of ISOS. Additionally, spiritual counselors and artists met at the start and end of the co-creation process to inform each other on the content of all meetings. Spiritual counselors advised artists on apparent vulnerabilities in patients. Spiritual counselors and the main researcher were available for artists to consult if needed. At the start of ISOS the artists and spiritual counselors attended four trainings of three hours each, developed by the multidisciplinary research team and led by the head of the department of Medical Oncology (Amsterdam Medical Centers), focusing on the needs of patients with advanced cancer. The embedding of patients, artists and spiritual counselors was also supported by the main researcher, a clinical psychologist (MSc), art-therapist (BA) and spiritual counselor (MA), who attended all supervision meetings of the artists with the two faculty members of the University of Arts Utrecht, and all meetings between spiritual counselors and artists. She also met regularly with the spiritual counselors and had screening interviews with all patients to establish feasibility of the project.

Patients were preselected by their attending oncologist and/or nurse, who informed patients about the ISOS project, its aims and general setup including the various elements of ISOS and possible challenges for patients. Patients who showed an interest for ISOS were contacted for a screening interview by the main researcher. Patients were further informed about the project, nature, number and duration of meetings with the spiritual counselors and artists, expected duration of the project, possible challenges (including emergence of intense emotions) for patients and their families, and possibilities for additional support if needed or preferred. Patients were informed they could choose to have the meetings with the spiritual counselors and the artists in the hospital, at the patient’s home or at the artist’s professional art studio. Al of these measures were taken to provide optimal and safe care for all partaking in ISOS.

### 2.2. Elements of ISOS

#### 2.2.1. RE-LIFE questionnaire.

**Fig 2:**
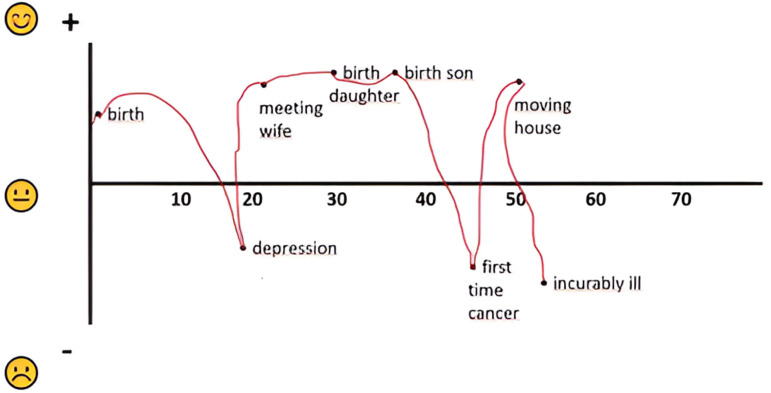
Case 1, high and low points of the life line drawn by the patient in the first RE-LIFE.

The self-reported RE-LIFE questionnaire [24] serves as a tool to deepen the understanding of the impact, assessment and interpretation of the strongest negative life event patients have experienced so far. A patient starts with drawing a life line from birth to the present moment by first identifying four to eight important events of which at least two are appraised as negative. The events are placed in a two-dimensional space defined by a horizontal time-line and a vertical axis indicating the relative appraisal (positive or negative) of the event. See Fig 2 as an example. The RE-LIFE further consists of nine scales of one to 15 items assessing patient’s experience of contingency, narrative meaning making, integration of the experience of contingency, current meaning of the life event, life goals, current attitude towards life, worldview, wellbeing, and, quality of life. See Hartog et al. [24] for a detailed description of the RE-LIFE. For this qualitative article we only present a description of the life line indicating patient’s appraisal of positive and negative life events.

#### 2.2.2. Rich Pictures.

Rich Pictures are a visual tool originating from systems engineering to explore complexity [25,33,34]. By making a drawing of a certain situation, a comprehensive overview of one’s experience of that situation can be expressed and communicated. In our research, Rich Pictures are drawings made by the patient in which they express experiences of contingency. The starting question for drawing the Rich Picture was, ‘Could you draw how the negative event you mentioned plays a role in your life now?’. This drawing can include all elements connected to and contributing to the experience [35,36]. See Bood et al [25]. for more in-depth information on Rich Pictures.

#### 2.2.3. Reading a story.

Patients received ten literary stories from the world literature, which had been edited for use in the ISOS project [19]. The exemplary stories illustrated various ways of dealing with experiences of contingency. During the meeting with the spiritual counselor, patients were encouraged to talk about 1) the main characters, 2) how they identified with the main characters, and 3) to make a connection between the story and their own life narrative. Then, their own life narrative was further discussed. See Kamp et al [19]. for further information on the reading of the selected stories.

#### 2.2.4. Co-creation process.

The artists used various entry points to collect and create inspiration for the patient, for instance at their art studio, in nature, or at the home of the patient. The artist stimulated the patient to explore materials that interested the patient while simultaneously linking these materials – and how the patient experienced these materials – to the patient’s life story. Patients were then encouraged to express this in a variety of initial art forms. Artists used these initial art forms as an inspiration to explore new avenues of artistic expression and created new artforms themselves which they introduced to the patients. Patients then reacted to and reflected on the artforms the artist had created and could create new artforms themselves or give feedback to the artist. After one or several rounds of this process the artist created the final work of art with high artistic value. While working with, and reflecting upon the created art, experiences of contingency were explored [20,26,30,31].

#### 2.2.5. Third meeting with the spiritual counselor.

The aim of this meeting was to support patients to gain more insight into the changes that may have occurred in their life narratives throughout participating in the ISOS project. The initiating question for this meeting was: ‘Looking back to the first meeting up to the final meeting, what has been most significant regarding what you have discovered about yourself?’.

#### 2.2.6. Final interview.

The researcher conducted a final interview with the patients to evaluate the entire project focusing on how they had experienced the project, their collaboration with the artist and the spiritual counselor, how they perceived the meetings of ISOS, what the meaning of ISOS as a whole was for them, and if they would recommend ISOS to others.

### 2.3. Data collection

The completed RE-LIFE questionnaires and the Rich Pictures were collected by the spiritual counselors and sent to the researchers. The meetings with the spiritual counselors (discussing the RE-LIFE, drawing Rich Pictures and discussing these, reading and discussing a story, and reflecting on the project), the artists (the co-creation process) and the researcher (final interview), were audio recorded and sent to the researchers.

### 2.4. Data analysis

We used a phenomenological approach to search for the lived experience of how patients moved through ISOS. We aimed to describe what patients experienced and how they experienced this by using a descriptive or transcendental phenomenological approach inspired by Husserl [37]. The aim of this approach is to distance subjectivity from the data collection and data interpretation. In this process, called epoche or bracketing, the researcher aims to set aside previous understandings, notions or interpretations to arrive at an understanding of what and how the patients have experienced the phenomenon [37]. Phenomenological approaches are particularly suitable to deepen understanding of human experiences by looking at the phenomenon through intensified engagement with the material, resulting in an increased understanding of the material [37]. The audio recordings of all meetings were imported in AtlasTi [38] and primarily analyzed by YW (MA, MSc, MSc, female) who has a professional background in art therapy, clinical psychology and spiritual care. By careful listening and extensive relistening to the full set of audio recordings of all meetings YW familiarized herself with the data. Subsequently, for each meeting, the most important, salient, emotional meaningful and rich parts were distinguished and were highlighted and subsequently extensively relistened to. Relevant quotations were selected. These quotations were presented in a chronological order following the elements of ISOS to illustrate how patients went through a process of integration of experiences of contingency during ISOS. All data analyses were discussed with both MSR (Associate professor, PhD, male), who has a professional background in religious studies, theology, spiritual care and qualitative and quantitative research and HvL (Professor, MD, PhD, PhD, female), who has a professional background in medical oncology, theology, qualitative and quantitative research. The original language of the ISOS project was Dutch. All translations into English for the current study were made by YW.

### 2.5. Case descriptions

We present two participants, who were the first to complete all of the meetings of the ISOS project, to illustrate how they integrated their experience of contingency into their life narrative.

### 2.6. Ethics

The study was exempted from ethical approval by the Medical Ethics Review Committee of the Academic Medical Center as the Medical Research Involving Human Subjects Act was not applicable (reference number: W20_436 # 20.483). The study complies with the Helsinki declaration and written informed consent was obtained from each participating patient, spiritual counselor and artist at the start of their enrollment. The written consent was subsequently securely digitally stored at the Amsterdam UMC deposit by YW. Patients were informed that they could withdraw and stop their participation in the current study at any moment without giving any reason. Patients were informed of the availability of the spiritual counselor for further support and that, if needed, referral to a psychologist at the hospital was possible. Also, they could cease meetings at any moment at their convenience.

## 3. Results

### 3.1. Participants and data collection

The two patient trajectories took place between January 2022 and August 2022, at the hospital where the spiritual counselor was employed, the patient’s home and the professional artist’s studio. The three meetings with the spiritual counselor lasted between 60 and 90 minutes, with a median of 70 minutes. Patients had six to eight art making meetings with the professional artist with a median of seven meetings. The duration of these meetings was 90–120 minutes. The meeting with the researcher lasted 60 minutes for both participants.

### 3.2. Case description One, the elements of ISOS

The first case was a 50-year-old male patient, who was undergoing treatment for bone cancer, which had metastasized to the lungs one year earlier. Five years prior to participating in ISOS, he was first diagnosed with cancer in his hip bone. The main experience of contingency he addressed was being diagnosed and living with incurable cancer.

#### 3.2.1. First meeting with the spiritual counselor – RE-LIFE and Rich Picture.

The low points the patient drew in the RE-LIFE life line (see Fig 2 for high and low points) were: a depression when he was a student, being diagnosed with bone cancer five years earlier, and being currently incurably ill. The high points were: being born, meeting his wife, the births of his two children and moving to the home of his dreams.Fig 2. Case 1, high and ....

In the RE-LIFE the patient described hearing the news that he was incurably ill as the most negative life event of his life: *“It was a blow when my doctor called while I was working alone at home. I was feeling numbed and I could not share it. Everything is different now, everything is focused on my departure.”*

The patient talked with the spiritual counselor about his (lack of) future perspective, acknowledging the loss of purpose and life goals and the need to search for new attainable life goals:

*“The chemotherapy did not work, my life expectancy is between one to eight years, and the cancer has metastasized. I am waiting for the situation to get worse. My most important goal to reach is to end my life in a dignified manner. I have no perspective in life. However, I do have an action perspective for my children: I want to be an example to them. Now I want to take care of my family the best I can. I want to pass on my way of living to them. I feel anger and sadness. My health is so overwhelming now that I can’t enjoy life. I also thought to myself that maybe it’s better to die soon, so my wife has time to find someone else. I want to prevent my children from being confronted with the disease process. On the other hand, what kind of goals did I have when I did not know I was ill? I don’t believe in anything, maybe it is easier to believe, because then I could hope for something.”*

**Fig 3:**
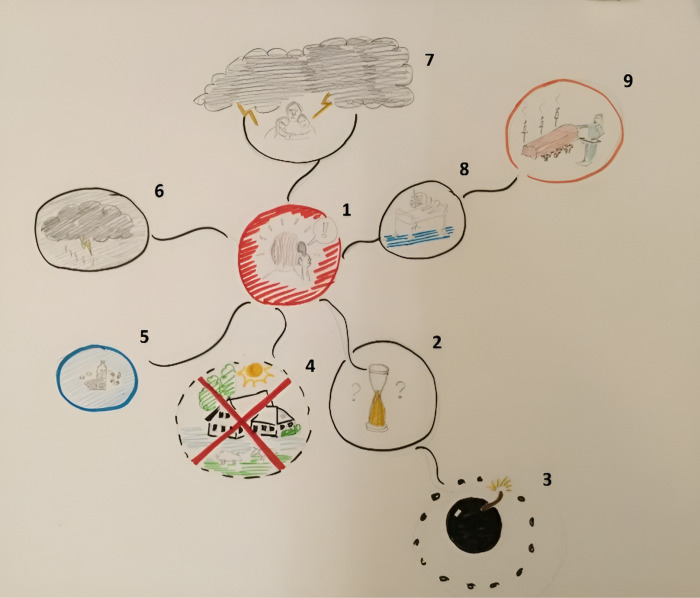
Case 1, the first Rich Picture. Numbers have been added as a reading guide and indicate the chronological order of the events depicted.

The patient’s Rich Picture showed different aspects of the impact of the negative life event (see Fig 3). As the patient described:

*“The first drawing is of receiving the phone call from my specialist telling me the news of being incurably ill. I experienced fright and numbness. The second drawing is a sand glass with question marks. This is related to the uncertainty surrounding the disease trajectory. The third drawing is a bomb which is going to burst sooner or later, and I am thinking about it a lot. The fourth drawing shows a farm with sheep that we bought, the home of our dreams. We sold it after hearing the news, as the money had to be put in more responsible things like paying off the student loans of my children. The dotted line depicts that thoughts about our dream house are slowly fading away. The fifth drawing shows all the medication I had to take during this disease trajectory. The sixth drawing shows dark clouds hanging above my head, and the seventh drawing shows my wife and two children, the clouds are darker than I could ever draw, and the meaning is sadness. Feelings of heaviness that at a certain point I can’t take care of my family any longer. The eighth drawing shows me lying in the hospital before I die, and the last [ninth] drawing is my funeral. I have depicted the drawings in the chronological order of how the illness will unfold.*

*When I would not have a family I may have looked around in the world to find some cure. Now my focus is on taking care of my family. In my twenties I had a depression, and what healed it was the perspective of creating a family. Now I have to let my family go.”*

#### 3.2.2. Second meeting with the spiritual counselor – discussing a chosen story.

Of ten available stories, the patient chose the story ‘The Ant’s Departure’, written by the Dutch author Toon Tellegen. The story revolves around the sudden departure of an ant and how the other animals in the woods respond to his disappearance. The patient had chosen this story because it reminded him of his love for his family. The patient explained:

“*You know the ant only through the stories of others. It reminds me of what I mean for other people and how I live on. Of all the animals, Squirrel and Hedgehog are the main characters who cannot let go of their grief. My wife looks like the squirrel; no one is as sad as she is. I want to know for sure that she can find happiness again when I am gone. I find it difficult to accept peaks because they only increase the feeling of wanting to hold on to life. My values are to care, to love, and to adopt an active approach. I feel a duty to take care of my legacy.*

#### 3.2.3. Co-creation process with a multi-disciplinary artist.

During the co-creation process, the patient and the artist met for six art making meetings in a time period of two months. The co-creation started with looking at pictures, so as to search for what interested the patient.

**Fig 4:**
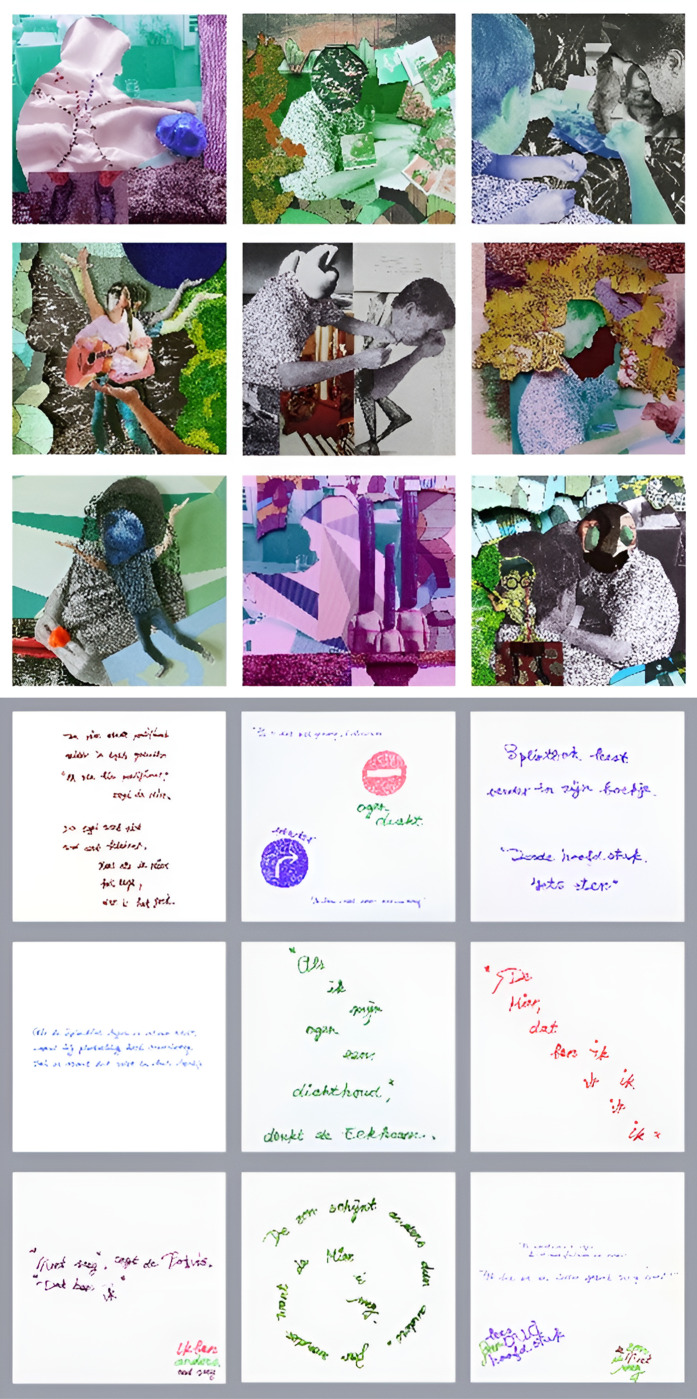
Case 1, Co-creation, the pictures and written texts forming the collages.

The idea arose to compile a collage. In his living room he already had a frame with his favorite vinyl records hanging on a wall. The story of Toon Tellegen about the departure of the ant was still in the back of his mind because of the variety in how the animals dealt with the loss of their beloved friend. The idea was developed to connect the chosen pictures with certain phrases of the animals in the story. The artist subsequently created nine collages. The patient was surprised and positively overwhelmed by her creation of the collages. He chose nine phrases from the story of the Ant and rewrote these phrases together with the artist in order to fit the collages. These texts were written on the backside of the collages by hand using a special pen that created three dimensional lines. All texts have their own shape according to the meaning of the text. The nine pictures each have the size of a vinyl album, their positions can be changed in the frame, and can be combined with the nine texts (see Fig 4 and Table 1). The patient recognized all kinds of aspects of himself in these complex pictures. The patient explained:

**Table 1:** Case 1, the nine texts that accompany the nine collages.

1. The Ant, that’s me	2. The sun is shining in a different manner, no wonder, because the Ant is gone	3. Now it’s enough Squirrel, I am not gone for nothing
4. ‘Not gone, said the Sperm Whale, ‘that’s me’	5. ‘If I would just close my eyes’ the Squirrel thinks	6. The Ant is the center of Hedgehog’s mind. “I am right here pontifical” the Ant says. The Hedgehog doesn’t know what that means but if the Ant says so, it should be okay
7. Splintbok^¹^ reads on in his booklet ‘Third chapter: Eat something’	8. As Splintbok is nearly falling asleep he is overcome with sadness, although the booklet did not mention this	9. The Cricket calls softly- it’s more of a whisper than calling: ‘After all, I am still here’

¹ Splintbok is a fantasy name of a non-existing creature.

*“The pictures connect to aspects of my life. In every picture the past and present moment are depicted. They also reflect my tendency to organize things in a systematic way. I was focused on past and future, but the artist focused on the present. I initially felt resistance to ascribe words to visual art-that’s not real art, I had to open up to also include texts because they actually support the pictures. Slowing down is not my normal way of being, activities have to be useful. Creativity is the first thing that falls apart in this disease process. I fled into behaving hectically, to stay in control.*

*I am milder now. If you are more open, you also receive more in return. My most important value is to take care of my family. However, all those years I wasn’t at home during the weekends because of all kinds of duties. Now I can finally be at home with my family during the weekends. The work is called ‘Nova’ which means new things, the start of an era. I am not being pulled into sadness anymore. The work shows that I was here, and I still am. This is the essence of creating meaning, it is like a portrait of me. Initially I thought of making a work of art that represented my pain, a work of art that showed me and my wife being separated by death. That idea changed to the present moment and what is of highest value now.”*

#### 3.2.4. Third meeting with the spiritual counselor – integration and second Rich Picture.

The patient explained to the spiritual counselor how he now deals with the experience of contingency, what his most important insights are and how he looks forward to life:

*“The co-creation meetings each lasted up to two hours, I had no idea where the co-creation would go, I surrendered to our joint creativity. I still worry about the future, but I think it will turn out well. Previously, everything had to go fast, now I think about living more slowly. I want to live according to my values. One of my values is hope, hope for something that is still possible in being able to live according to my life goals. I choose not to travel the world in search for a cure. An important goal is caring for others, but other goals are now connected to this, in proportion to it. The co-creation product is about values. Now my most important value is that everything is in balance. I have found qualities I didn’t know I had, like patience and creativity. I see my wife developing, we look at the work of art together and talk about it. Everything was about my family, but there are more things in life, a life besides being ill, space for other things. Normally I want to take control, but now I let it go. In the beginning there was sadness but now I see that all the elements in the collage have its place in life. I don’t have hope for a cure but you can cut hope into smaller segments, for example hope for a good week. Like in the story of the ant, ‘now it is enough, it is time to eat’. When I was on holiday a woman prayed for me in a church I visited. She said: “you have to know you are loved”. I translate this to mercy and charity. I feel a bit like a sailor in the storm who starts to pray to God in times of trouble. Maybe I am a little bit more open to another perspective.”*

**Fig 5:**
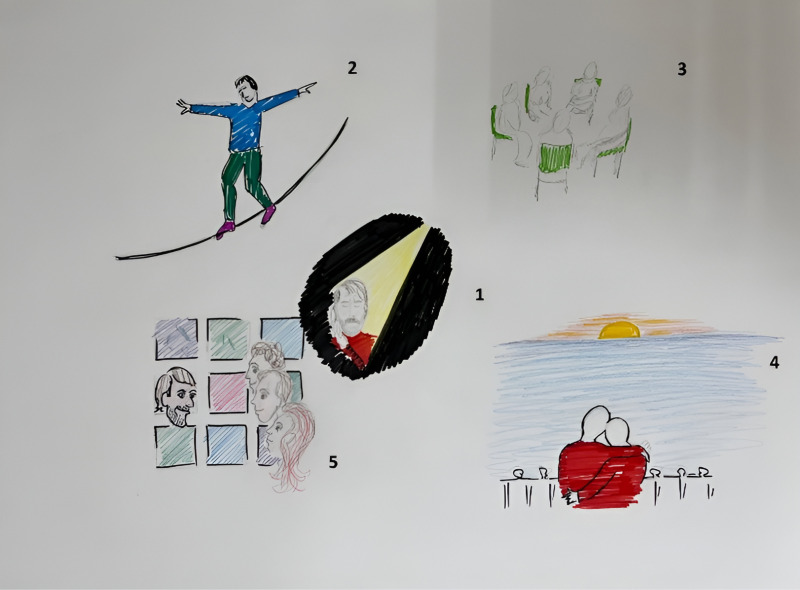
Case 1, the second Rich Picture. Numbers have been added as a reading guide and indicate the chronological order of the events depicted.

Then, the patient continued to draw his second Rich Picture (see Fig 5). The patient described:

*“The middle picture depicts the same message: you are incurably ill. But now I see there is a crack in everything, that’s where the light gets in. The sadness and shock is still here but there is a bit of light shining through. Being ill remains unreal, but it is not all encompassing anymore. In the first picture I have depicted myself from the front instead of sideways. The next picture shows that I feel I am balancing on a cord, my hands are spread to create balance between sadness and a valuable life, my mouth is laughing. At the moment my life is about balancing on a string. The third picture shows my cancer survivors support group, which helps but is also confronting because someone has just died. In the fourth picture I have drawn myself and my wife at the ocean, which could be the last time together. The ocean is empty and peaceful. Because we moved houses we have more time to travel now. Our heads are leaning against each other, the illness brings us closer together, not always enough but more than in the past. We have an outlook towards my life’s end, the sun is slowly going down, it’s not falling out of the sky, I hope it will be the same for me. The fifth picture shows the collage framework and the family together. The framework is hanging centrally on the wall in our house. It is about me but also about my family, this generates topics for conversation. I wanted to express our eye contact. Contact is important. After hearing the news we hardly talked about it, now it is more open, we have more breathing room. The colors are beautiful and it radiates cheerfulness, we are not sad.”*

#### 3.2.5. Evaluation meeting with the researcher.

The patient reflected on the ISOS project together with the researcher. He spoke about what the project had brought him:

*“I have become aware that I was mostly hurrying through life. During the project I have realized that I can also make something happen in an organic way. These different aspects of myself were also part of the story of the Ant. I started the project to please my oncologist because I was feeling depressed, but in hindsight I have really gotten something out of it. It has enriched me. I would suggest to do this together with a partner and children, that’s what I did, by talking about it after each meeting and receiving input from them. In this way they will also recognize something of themselves in the work of art.”*

### 3.3. Case description Two, the elements of ISOS

The second case-study focuses on a 64-year-old female patient, who was diagnosed with breast cancer five years earlier, which had currently metastasized to several body areas. She was undergoing chemotherapy at the moment of participation. The main experience of contingency she addressed was a combination of her own disease and her (step)daughter’s disease, who died from metastasized cervical cancer.

#### 3.3.1. First meeting with the spiritual counselor – RE-LIFE and Rich Picture.

**Fig 6:**
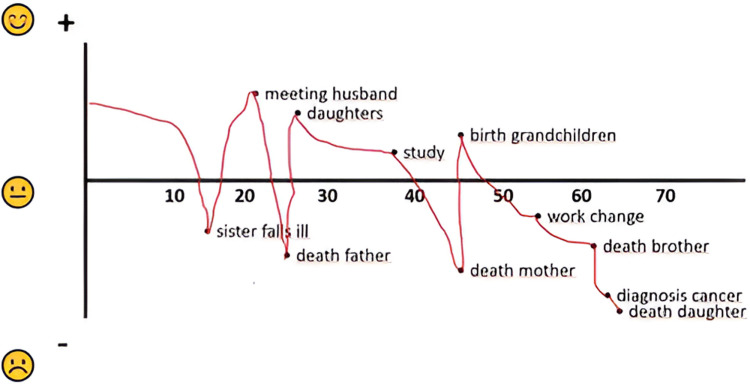
Case 2, high and low points of the life line drawn by the patient in the first RE-LIFE.

In this first meeting, the patient discussed the way she had drawn the RE-LIFE life line (see Fig 6), how she had filled out the RE-LIFE questionnaire, and drew and discussed a Rich Picture. The low points the patient drew in the RE-LIFE were: her sister falling ill with schizophrenia when she was an adolescent, the death of her father, mother, and brother, a change in her work, and her (step)daughter being diagnosed with metastasized cervical cancer. The patient subsequently received the diagnosis of metastasized breast cancer herself and during that period her (step)daughter died of her disease. The high points the patient drew were: meeting her husband, receiving her (step)daughters, completing a study, and the birth of her grandchildren.

The patient described the most negative life event being her (step)daughter’s disease combined with her own cancer diagnosis. As the patient described:

*“Every pitfall in life has taught me things, I am aware there are boundaries, but it has made me stronger, this illness is just bad luck – I have a humanistic approach to life. Right now I am increasingly starting to think about the end of my life, but I also find it difficult to think about it. I feel I need to prepare my environment for my death. To be able to create a good end of life is an important life goal, but I want to postpone it. I experience a dilemma between my environment, which is not aware that I feel I am nearing the end and doesn’t want to talk about it, and my own feelings, physical pain and tiredness. I have been ill for 5 years now, and over the years the illness has been pushed to the background. I worked many years as a psychologist and experienced difficulty letting go of my job because I like solving problems for people. My sister calls me several times a day. My other daughter recently said: “We don’t know what to do when you would fall away”. I am afraid to talk about the future, my husband can’t deal with conversations about my nearing death. I am thinking a lot about how my daughter lived in the last period of her life and why she chose for euthanasia, she left two young children behind and had lots of pain and increasing disability. I feel I am in an environment where I support others but there is little support for me. I would like to have assisted end of life care, and arranged this with my GP. I was afraid I would not be able to arrange this once my health really would start to deteriorate.”*

**Fig 7:**
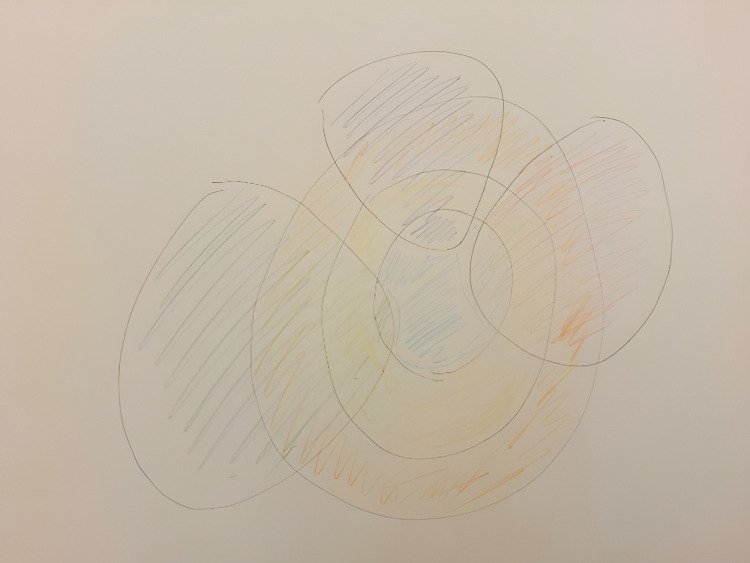
Case 2, the first Rich Picture.

The patient’s Rich Picture showed different aspects of her current life, including the impact of the negative life event (see Fig 7). As the patient explained:

*“I did not want to draw in a figurative way, I was surprised by the question to draw something. The blue circle in the middle is me, the other circles penetrate through me, and have an impact on me. They hinder my sense of wholeness. These aspects could be my mother, my brother, my work, and the illness. The yellow are the people around me, my connections, and the orange is how I have lived. The green – let’s call that the disease. It has impact but most of me is still whole. I am avoidant of it, but it is here. I am consciously wanting to keep it small, the here and now is most important, that is what I want to focus on. Maybe I should have made it look more threatening, but I did not think about that. The purple is my old mother, but that is too interpretative. It is not a heavy drawing, I am not so outspoken, I am mild, I don’t have so much negative feelings, that is my way, my life.”*

#### 3.3.2. Second meeting with the spiritual counselor – discussing a chosen story.

Out of ten available stories the patient chose the story ‘Code Catnip’ written by the Dutch author Jacques Vriens. The story revolves around a grandfather who is ill, and his relationship with his grandson. The grandfather has lung cancer and the family is very sad about it. The grandfather does not want to accept that he is ill or is going to die. As the patient explains:

*“I had to cry when I read it and talking about it makes me cry again now. I was deeply touched by it. It opened up my emotions. We don’t talk about the process of dying, they all want to hold on to me, and keep going, and that is a denial. I am not ready to confront my environment, it is like they don’t realize how ill I am. To keep going I make it small. I can well imagine that the family in this story is reacting the way they do, and that the grandfather also wants to keep it at bay. I am introvert and don’t show my sadness. Making the end visible is what this story is about. I just don’t want to die, but it is a process that is moving into another phase now. I am surprised that I allow this now, I cannot deny it any longer. I don’t want to feel the powerlessness of being unable to take care of myself and instead being cared for. My husband says I am doing too many things, and I am, in reaction to him, trying to pretend that I am feeling well. My daughter resisted being cared for, and the family said if you want to go, then go, and then she died.”*

#### 3.3.3. Co-creation process with a visual artist.

**Fig 8:**
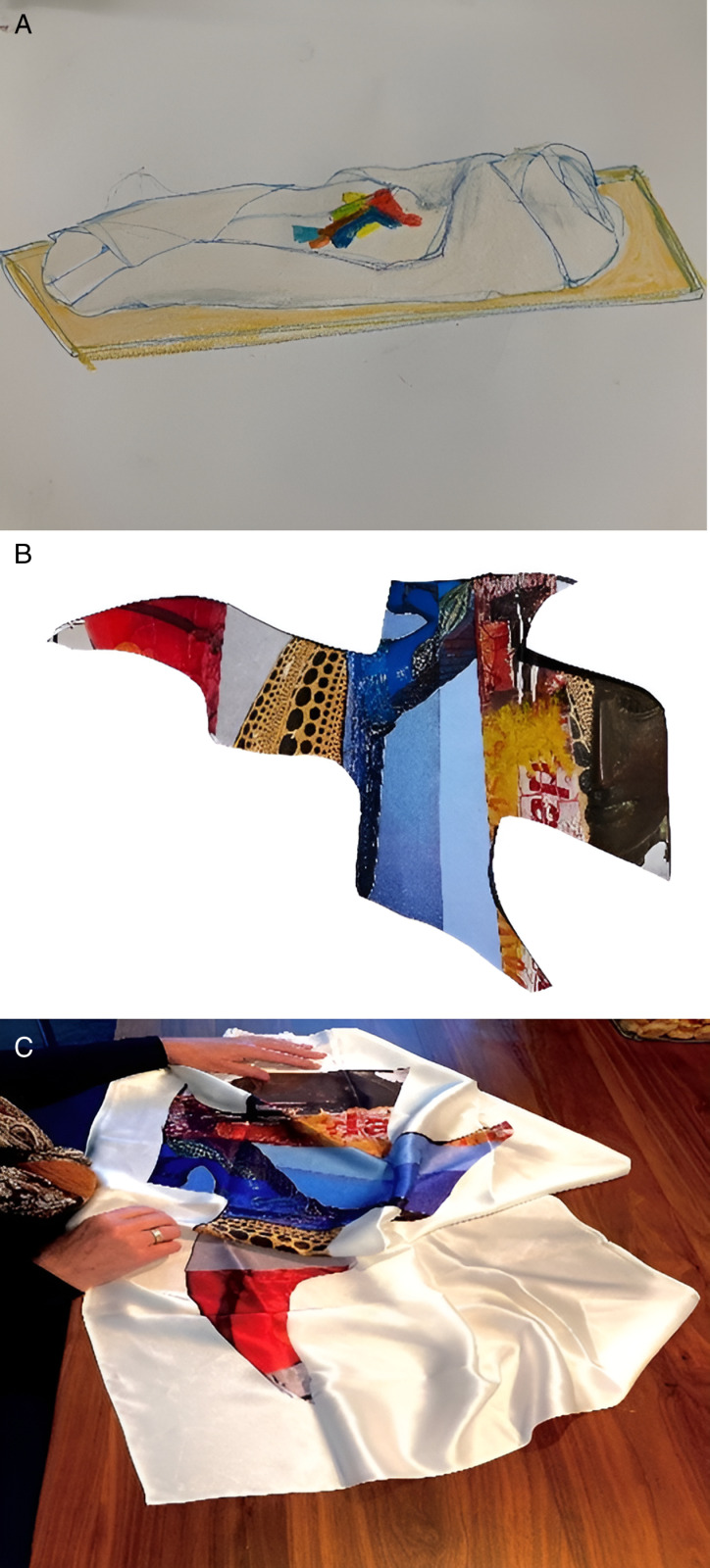
Case two, Co-creation. From left to right: drawing of a burial garment, the image of the Bird of Paradise, the white satin scarf with a print of the Bird of Paradise.

The patient and artist met for eight art making meetings in a time period of five months. Before the co-creation meetings started the idea arose to make a burial garment (see Fig 8). The co-creation process was initiated with wax pastels and watercolor crayons. The patient brought pictures of specific things that were important to her and talked about elements of her life story. She cut up the pictures and experimented with making color compositions. The arrangement of pieces of pictures started to look like a bird, which the patient named ‘A Bird of Paradise’ (see Fig 8). She gradually changed her mind about creating a burial garment as she had noticed that her husband was not yet ready for her to order a burial garment. Instead she decided she wanted to create a scarf that could be folded into the burial garment. She decided it would be a white satin scarf with the Bird of Paradise printed on it (see Fig 8). She also created a booklet with pictures of the co-creation process with the intent to open up the communication with her family about the process of dying. She mentioned that Leonard Cohen wrote a song (Anthem) where he sings: “There is a crack in everything, that’s where the light gets in” and wanted to add this text to the booklet. In one of the last art making meetings she took her husband to the studio to show what she had made so far. He reacted that he was afraid she was going to order the burial garment and shortly after would die. In the last art making meeting the artist travelled to the patient’s home and presented the scarf to her, while her husband was also present. Together they reflected on the co-creation process, the scarf and the booklet. The patient explained:

*“The images, stories and memories are present in this scarf, is has been a process of placing and naming things. I am normally a rational person, but this was creative and gave me the chance to move through barriers I would otherwise not have been confronted with. The person who watches the image is enabled to influence the meaning of the image, it is a beautiful thought that others can create their own meaning around it. I am going to give this to my children and grandchildren. It does get more clear this way (gives husband a hug) I understand it has touched you.”*

*Her husband responded: “I find the scarf very beautiful but you do have to prepare the children before giving this booklet. I don’t feel an inclination to let this beautiful scarf be burned in the oven, I might frame it and hang it on a wall.”*

*She continued: “The burial garment also needs to cover my head, and then only the symbol on the scarf is visible. I want to be taken from my home straight to the crematorium to be burned. The soul needs to let go of the material world. The symbolic meaning of the bird is that it expresses positivity.*

*I don’t want to be ill, but the reality is that I am. I have been writing my last will, and have been thinking about the funeral.”*

#### 3.3.4. Third meeting with the spiritual counselor – integration and second Rich Picture.

The patient explained to the spiritual counselor how she now perceived dealing with the experience of contingency, what her most important insights were and how she looked forward in life. The patient explained:

*“I find the steps in this project manageable, to break down my process into parts and have a visual reflection of the next step, in this way I can oversee the whole process. Several stories of my life also passed by, it was not a rational process. There is going to be a moment where I am also going to show this to my family, I am afraid of their emotions. My grandson had recently said to a family member: “I will start studying again and then my grandmother will die, and then what?” He might fear a similar situation as when his mother died. I also want to give my grandchildren some light, I do talk about it with them in a jokingly way, the thought of the bird flying away.”*

**Fig 9:**
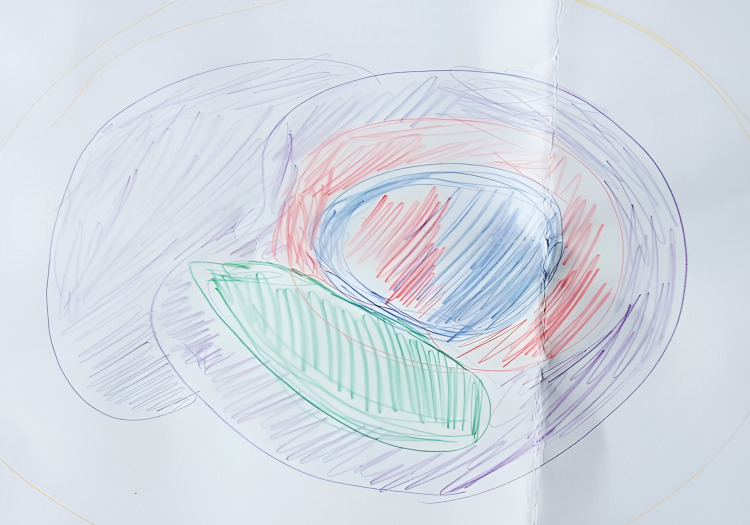
Case 2, second Rich Picture.

Accordingly, the patient continued to draw her second Rich Picture (see Fig 9). As the patient described:

*“There is a shield around me, and it has been opened up by this whole project. Those colors are the others surrounding me. Sharing emotions brings more trouble than ease to me. I am guilty of that myself, because I do this myself, not to speak about it. I receive text messages saying ‘you are so strong’, thereby creating a perpetuating cycle. My husband found it very difficult that I started to talk about the burial garment. I also worry about the grandchildren, my grandson blocking his life because yet another difficult period is about to come. We now do share beautiful things, but regarding my departure it is still difficult to speak about. It is that powerless feeling that I can only help them in a minimal way.”*

#### 3.3.5. Evaluation meeting with the researcher.

During the evaluation meeting with the researcher the patient reflected on the ISOS project and elaborated on what the project had brought her:

*“In preparation for every meeting it was on my mind, because I found it important. The stories that are in me, the images, conversing about it, I found this really nice to do. I think it can add something for others too to become aware of your process like this. The grade for advising it to others would be a nine because I think it is a gift and it would be great if this would be added to end of life care. Because of this project I am more focused on being in the here and now. I am still positive about life, there isn’t a moment when I think it should be over. Initially I was avoidant to start with this process, but from the second meeting with the spiritual counselor onwards it did make me think about preparing for my death. The story ‘Code catnip’ made me think about how my environment is dealing with this, I try to be more open, they should also know that it is ending. I feel more concrete, more thoughtful, I do move towards acceptance. My daughter chose for euthanasia, if life is not bearable anymore I want to be able to take that step, and this project has made me more aware of that. The idea of actually ordering the burial garment is somewhat confronting still. Between me and my husband the scarf is like a middle ground, which can be used at the funeral. The disease process generates new hope continuously, but this is the palliative stage and the awareness that this is ending also has become greater. The process of my daughter’s end of life choices did have an active role in these past months. She has become a greater part of my thinking. The fact that my grandson can’t make a decision has made me aware that I remain to be sensitive towards my environment and care for them. The meaning of the bird, lots of color, I really love birds and our daughter had said, “when you see a Robin you must think of me”. They have a higher purpose. The bird has been compiled out of photos of my environment, memories and colors. The idea that one can fly away appeals to me.”*

## 4. Discussion

This article describes the experiences of patients with advanced cancer during integration of experiences of contingency within the setting of the ISOS project. These two cases illustrated how a process of integration of experiences of contingency was initiated and may unfold.

Both patients were trying to fulfill life goals in relation to their family. For the patient in case one this revolved around family becoming more important for him and investing more time in his family. The patient in case two encountered the challenge that her partner and children were not yet able to accept her immanent death. Both patients succeeded in achieving their family goals despite the obstacles encountered. These goals encompassed connection to loved ones, currently supporting loved ones and continuing supporting them also after their death.

Although not originally intended, for both patients the created work of art also served as a legacy, in particular for their families. A legacy is described as “the process of leaving something behind” [39], which is “a means of reflecting the existential or spiritual realms of an individual’s life experiences” [39]. Creating a legacy supported the realization of patient’s life goals in the future [40,41]. Both patients created their works of art for their family with an implicit intent to support the ones left behind.

Patients’ participation in ISOS initiated the unfolding of a process of contemplation and a movement towards more acceptance of their nearing death. The co-creation process invited the patients to not only talk about their experiences, but also created space to sense and work with materials. The work of art served as a ‘felt sense’ instrument in the interaction between the patient and their environment. In the first patient working with pictures helped him to focus on his present relation to his environment instead of dwelling in the past or worrying about future events. This patient described that he experienced more peace and less negative emotions despite experiencing the vulnerability of his contingent situation. It seems that patient two needed more time to renegotiate her needs with her family. The scarf was a communication tool between this patient and her husband and subsequently her family. Working with the material enabled her to step by step communicate her experience. Also this patient reported increasing contemplation and peace with her illness. These two cases indicate that the integration of experience of contingency could be a mutual development between the patient and his/her environment.

ISOS was not only headed up by a multidisciplinary team, but the project evolved into a transdisciplinary endeavor [42]. This transformation occurred as the project’s objectives and methodology transcended mere knowledge and experience exchange, aiming to integrate diverse disciplines into a cohesive, holistic approach. The transdisciplinary nature of ISOS fostered the creation of a unified intellectual framework that applied across multiple domains, encouraging collaborative problem-solving and innovation beyond traditional disciplinary boundaries. Indeed, the aim of transdisciplinarity is to use boundaries of disciplines and to combine or cross these boundaries to come to a deeper understanding of phenomena. Crucial in this approach is to include practice oriented disciplines as the practical execution of concepts and models reveals how such ideas translate into practicality, but also which limitations accompany a practical implementation [43,44].

In contrast to several psychological interventions including art therapy, ISOS did not aim to treat psychopathology or strive after specific therapeutic outcomes [45–47]. ISOS entailed an existential approach originating from spiritual care. The integration of art as an additional discipline served to enhance the incorporation of contingency experiences into individuals’ life narratives. Based on the input of patients, artists made the work of art. This approach provided a balance between exploration through the senses and the life narrative by the patient, combined with the production of an aesthetic work of art by the artist. In some cases the artist made the complete work of art with verbal input from the patient, in other cases the patient made some parts of the work of art. Yet, the artist was responsible for ensuring the aesthetic value of the work of art. We do not know what the outcomes, such as engagement, exploration and expressing deeper meanings, would have been if the work of art was entirely made by the patient, as is more common in, for example, art therapy. The current study suggests that involvement of a professional artist can support a high level of distancing, which supports patients in the integration of the experience of contingency in their life narrative [29]. Indeed, the introduction of the final work of art by the artist to the patient invited another level of reflection, which further supported the integration of the experience of contingency.

Vulnerabilities could arise when patients explored their life narrative. To support patients, the spiritual counselors were professionally equipped to address these vulnerabilities with care and adequacy. The professional artist addressed potential issues during the supervision meetings with the spiritual counselor and the main researcher, and could consult both if needed.

ISOS did not provide support to the patients’ families and their direct environment. For future implementation it may be beneficial to provide such support and offer possibilities for participation in the project.

Some patients experienced logistic limitations and thus home visits were provided. However, meeting at the home of the patient could create a vulnerable setting as the artist or spiritual counselor was a visitor and the patient became a host. The home setting is more private than a hospital setting and could be experienced as too exposing for patients. Additionally the presence of family members could also be a disturbance, especially when patient’s and family members’ experiences were not aligned, as for instance in case description two [48]. To support patients, formal training at the start of ISOS covered sensitivity of working at a patient’s home, including the presence of family members. Special care was taken to minimize disruption by family members during meetings with the spiritual counselor or professional artist. To prevent prolonged duration of the meetings, which could easily happen in a home setting, start and end times were preset. Patients were encouraged to communicate their needs and could end meetings at any time without further explanation. Allowing meetings to take place in a home setting proved to work well for patients.

This article provides preliminary insight into how the combined elements of ISOS help patients to explore their life narrative and to deal with the challenges that arise in the face of advanced cancer. Although the two case studies yielded rich information, the findings cannot be generalized to other patients within ISOS, let alone other groups of patients. We still need to analyze the data of the other patients within ISOS to determine the variety in the processes of integration of experiences of contingency into the life narrative. Future research is also needed to identify which themes are relevant for patients and if there are recurring, communal themes. Other avenues for future research include investigating whether focusing on life goals is a prerequisite for integration of experiences of contingency, or, what the importance and effect is of the work of art as a legacy.

## 5. Conclusion

The current study provides preliminary insight into how patients can go through a process of integration of experiences of contingency into their life narrative, which could inform the development of future support for patients with advanced cancer dealing with experiences of contingency. Specifically, offering patients possibilities to express themselves through materials within an artistic setting could support these patients to find new words and additional non-linguistic ways of expressing their experiences, and thereby facilitate the integration of experiences of contingency into their life narrative.
